# Untargeted Metabolomics Reveals Distinct Metabolic Signatures of Lactic Acid Bacteria in Food Fermentation and the Same Pipeline Applied to Foodborne Pathogen Detection

**DOI:** 10.3390/metabo16070513

**Published:** 2026-07-22

**Authors:** Hao Li, Yuchen Tao

**Affiliations:** School of Food Engineering, Maanshan Teacher’s College, Ma’anshan 243041, China

**Keywords:** metabolomics pipeline, lactic acid bacteria, food fermentation, foodborne pathogens, biomarker discovery, GC-MS, UPLC-Q-TOF-MS, multivariate analysis

## Abstract

**Highlights:**

**What are the main findings?**

- Untargeted metabolomics (GC-MS and UPLC-Q-TOF-MS) revealed distinct species-specific metabolic signatures: *L. plantarum* favored organic acid production, while *L. rhamnosus* exhibited amino-acid-centric metabolism.
- Mixed-culture fermentation displayed complementary metabolic profiles, suggesting synergistic cross-feeding interactions between the two LAB strains.
- The same analytical pipeline, when applied to pathogen detection, achieved AUC values of 0.87–0.89 for three major foodborne pathogens (*E. coli* O157:H7, *S. enterica*, and *L. monocytogenes*) with detection times of 18–30 h, representing a substantial reduction compared to conventional culture methods (5–7 days).

**Abstract:**

**Background/Objectives**: Lactic acid bacteria (LAB) are essential drivers of food fermentation, yet systematic metabolic comparisons across different LAB strains remain underexplored. This study characterized and contrasted the metabolic fingerprints of *Lactiplantibacillus plantarum* and *Lacticaseibacillus rhamnosus* in vegetable fermentation and subsequently evaluated whether the same untargeted metabolomics pipeline could be applied to rapid foodborne pathogen detection. **Methods:** A multi-platform untargeted metabolomics strategy integrating GC-MS and UPLC-Q-TOF-MS was applied to profile four experimental conditions: non-fermented control, *L. plantarum* monoculture, *L. rhamnosus* monoculture, and mixed-culture fermentation. Multivariate statistical tools (PCA and PLS-DA) were used to identify differential metabolites and perturbed pathways. The identical analytical workflow was then applied to beef samples artificially contaminated with *Escherichia coli* O157:H7, *Salmonella enterica*, or *Listeria monocytogenes*. **Results:** Across all samples, 847 metabolites were annotated, of which 312 showed significant abundance changes upon fermentation. PLS-DA delivered robust group discrimination (R^2^X = 0.89, R^2^Y = 0.95, Q^2^ = 0.91). Organic acids (32.0%) and amino acids (24.0%) dominated the metabolic landscape, with lactic acid, acetic acid, diacetyl, and acetoin as the most elevated compounds. KEGG analysis highlighted glycolysis/gluconeogenesis, pyruvate metabolism, and branched-chain amino acid degradation as the most heavily rewired pathways. When the same pipeline was applied to pathogen detection, it yielded AUC values of 0.89, 0.87, and 0.88 for *E. coli* O157:H7, *S. enterica*, and *L. monocytogenes*, respectively, with detection times of 18 h, 24 h, and 30 h. **Conclusions**: This work delivers a side-by-side metabolic atlas of two prominent LAB species and demonstrates the technical portability of an untargeted metabolomics pipeline from a fermentation model system to a pathogen detection scenario. The identified biomarker panels warrant further validation in diverse food matrices, and translation to routine monitoring will require matrix-specific model training and validation.

## 1. Introduction

Fermentation stands as one of humanity’s oldest food-processing technologies, valued for its capacity to extend shelf life, elevate nutritional value, and enrich organoleptic qualities [1,2]. At the heart of most fermented foods lie lactic acid bacteria (LAB)—a polyphyletic group of Gram-positive organisms that drive carbohydrate conversion into organic acids, generate aroma-active volatiles, and produce bioactive metabolites with documented health benefits [3,4]. The metabolic output of LAB during fermentation is not merely a byproduct; it fundamentally determines the quality, safety, and consumer appeal of the final product.

Over the past decade, metabolomics has come of age as a key analytical discipline for profiling small molecules (<1500 Da) in complex biological matrices [5,6]. Positioned at the downstream end of the systems biology cascade, metabolomics captures the integrated phenotypic response to genomic, transcriptomic, and proteomic perturbations, offering a functional readout that is both sensitive and information-rich [7]. Its application to food science—often termed “food metabolomics”—has opened new windows into fermentation dynamics, biomarker discovery, and safety monitoring [8,9].

A central tenet of LAB fermentation is strain specificity. Different species—and even individual strains within a species—can produce markedly different metabolic profiles under ostensibly identical conditions [10,11]. *Lactiplantibacillus plantarum* and *Lacticaseibacillus rhamnosus* are two economically important LAB with distinct ecological niches and metabolic repertoires. *L. plantarum*, a facultative heterofermenter, is widely used in vegetable, dairy, and meat fermentations and is recognized for its versatile carbohydrate metabolism, bacteriocin production, and exopolysaccharide synthesis [12,13]. *L. rhamnosus*, by contrast, is best known for its probiotic properties and is frequently employed in dairy applications, where it contributes to both flavor development and bioactive peptide generation.

Despite considerable genomic and proteomic data for these species, a systematic, side-by-side metabolomic comparison of their fermentation profiles has been lacking [14,15]. Furthermore, while metabolomics has been applied separately to LAB fermentation and pathogen detection, few studies have evaluated whether a single standardized pipeline can perform robustly across both application domains. At the same time, metabolomics is gaining traction as a potential solution to a persistent challenge in food safety: the slow turnaround of conventional pathogen detection methods. Traditional culture-based approaches typically require 5–7 days for isolation and confirmation, creating a critical bottleneck in outbreak response and supply chain surveillance [16,17]. Metabolomics offers a conceptually appealing alternative—detection through pathogen-specific metabolic signatures, potentially within hours rather than days [18]. However, the extent to which a metabolomics workflow optimized for one food matrix can be transferred to another remains an open question that warrants systematic investigation.

The present study was designed around two parallel objectives. First, the study aimed to characterize and contrast the metabolic profiles of *L. plantarum* and *L. rhamnosus* in a model vegetable fermentation system, using untargeted metabolomics as the discovery platform, and to identify the key differential metabolites and metabolic pathways that distinguish these two species and their mixed-culture behavior. Second, we sought to evaluate whether the same analytical pipeline—employing identical extraction protocols, instrument parameters, and data processing workflows—could achieve satisfactory classification performance for rapid detection of three major foodborne pathogens (*E. coli* O157:H7, *S. enterica*, and *L. monocytogenes*) in contaminated beef. It is important to clarify that these two objectives are not presented as a single, unified biological application. Rather, they share a common methodological framework: the same untargeted metabolomics pipeline is benchmarked across two distinctly different food microbiology scenarios—a well-controlled fermentation model and a more complex pathogen detection matrix. This “pipeline portability benchmarking” design allows us to assess both the strengths and limitations of the workflow under contrasting conditions, providing practical reference data for laboratories considering a standardized metabolomics platform for multiple food-safety tasks.

## 2. Materials and Methods

### 2.1. Chemicals and Reagents

All chemicals and solvents were of analytical or LC-MS grade. Methanol, acetonitrile, and formic acid were sourced from Fisher Scientific (Waltham, MA, USA). Pyridine, methoxyamine hydrochloride, and N-methyl-N-(trimethylsilyl)trifluoroacetamide (MSTFA) were obtained from Sigma-Aldrich (St. Louis, MO, USA). Ultrapure water (18.2 MΩ·cm) was produced using a Milli-Q Direct 8 system (Millipore, Bedford, MA, USA).

### 2.2. Bacterial Strains and Culture Conditions

*L. plantarum* ATCC 14917 and *L. rhamnosus* ATCC 53103 were procured from the American Type Culture Collection (ATCC, Manassas, VA, USA). Stock cultures were preserved at −80 °C in MRS broth containing 20% (*v*/*v*) glycerol. Prior to experimentation, strains were subcultured twice in MRS broth at 37 °C for 18 h under anaerobic conditions (85% N_2_, 10% H_2_, and 5% CO_2_).

Pathogen strains—*E. coli* O157:H7 ATCC 43895, *S. enterica* subsp. *enterica* serovar Typhimurium ATCC 14028, and *L. monocytogenes* ATCC 19115—were cultured in tryptic soy broth (TSB) at 37 °C with orbital shaking (150 rpm) for 18–24 h.

### 2.3. Model Vegetable Fermentation

A standardized vegetable broth was prepared containing (per liter) glucose (20 g), peptone (10 g), yeast extract (5 g), NaCl (5 g), and a 1:1 (*v*/*v*) mixture of carrot and cabbage juice (200 mL). The vegetable juice was obtained by homogenizing fresh carrots and cabbage with distilled water (1:1, *w*/*v*), followed by filtration through cheesecloth. The final broth (pH 6.5) was autoclaved at 121 °C for 15 min.

### 2.4. Pathogen Inoculation and Enrichment

Vegetable fermentation model: Four treatment groups were established: (i) uninoculated control; (ii) *L. plantarum* (inoculum ~1 × 10^6^ CFU/mL); (iii) *L. rhamnosus* (~1 × 10^6^ CFU/mL); and (iv) mixed culture (5 × 10^5^ CFU/mL of each strain). Fermentation was carried out at 30 °C for 48 h, with six biological replicates per group. Sampling occurred at 0, 6, 12, 24, 36, and 48 h for subsequent metabolomic analysis.

Beef pathogen inoculation model: Minced beef (25 g portions) was spiked with each pathogen individually at approximately 1 × 10^2^ CFU/g; unspiked samples served as controls. Enrichment was performed in selective broths: Oxford broth for *L. monocytogenes*, modified Kauffmann broth (MKTTn) for *S. enterica*, and modified tryptone soy broth (mTSB) for *E. coli* O157:H7. Each pathogen inoculation condition was performed in six biological replicates (*n* = 6 per strain), with uninoculated beef samples serving as negative controls (*n* = 6). At 6, 12, 18, 24, and 30 h post-inoculation, 1 mL aliquots were withdrawn for metabolite extraction.

Methodological consistency and quality controls: Despite the matrix disparity between vegetable broth and beef, identical extraction and analytical protocols (Section 2.5) were applied across both experimental arms to ensure methodological consistency. Blank controls (selective enrichment broth only, without beef or pathogen) and negative controls (uninoculated beef samples processed identically) were included in each experimental batch to monitor for background contamination and instrument drift.

### 2.5. Metabolite Extraction

For GC-MS analysis, 100 mg of each sample was extracted with 1 mL of methanol–chloroform–water (2.5:1:1, *v*/*v*/*v*) spiked with 10 μg/mL ribitol (internal standard). The mixture was vortexed (30 s), sonicated (15 min, 4 °C), and centrifuged (12,000× *g*, 10 min, 4 °C). The supernatant (500 μL) was dried under nitrogen. Derivatization was performed in two steps: methoxyamination (80 μL of 20 mg/mL methoxyamine hydrochloride in pyridine, 37 °C, 90 min), followed by silylation (80 μL MSTFA, 37 °C, 30 min).

For UPLC-Q-TOF-MS, 100 mg of sample was extracted with 1 mL of methanol–water (4:1, *v*/*v*) containing 10 μg/mL L-2-chlorophenylalanine (internal standard). After vortexing (1 min), sonication (20 min, 4 °C), and centrifugation (12,000× *g*, 10 min, 4 °C), the supernatant was filtered through 0.22 μm PTFE membranes.

### 2.6. GC-MS Analysis

GC-MS analyses were conducted on an Agilent 7890B gas chromatograph coupled to a 5977B mass spectrometer (Agilent Technologies, Santa Clara, CA, USA), fitted with a DB-5MS capillary column (30 m × 0.25 mm × 0.25 μm). Helium (99.999%) was used as carrier gas at 1.0 mL/min constant flow. Splitless injections (1 μL) were performed. The oven program started at 70 °C (2 min hold), ramped to 180 °C at 10 °C/min, then to 280 °C at 5 °C/min, with a final hold of 8 min. Mass spectra were acquired in electron impact (EI) mode at 70 eV over *m*/*z* 50–650. Source and quadrupole temperatures were maintained at 230 °C and 150 °C, respectively.

### 2.7. UPLC-Q-TOF-MS Analysis

UPLC-Q-TOF-MS analysis was carried out on an ACQUITY UPLC system coupled to a Xevo G2-XS Q-TOF mass spectrometer (Waters Corporation, Milford, MA, USA). Chromatographic separation was achieved on an ACQUITY UPLC BEH C18 column (100 mm × 2.1 mm, 1.7 μm) at 40 °C. The mobile phase consisted of 0.1% formic acid in water (A) and acetonitrile (B), with the following gradient: 0–2 min, 5% B; 2–10 min, 5–40% B; 10–15 min, 40–70% B; 15–18 min, 70–95% B; 18–20 min, 95% B; 20–20.5 min, 95–5% B; 20.5–23 min, 5% B. The flow rate was 0.4 mL/min, and the injection volume was 5 μL.

Mass spectrometry was performed in both positive and negative electrospray ionization modes. Key parameters were capillary voltage 2.5 kV (ESI+) and 2.0 kV (ESI−); cone voltage 40 V; desolvation temperature 400 °C; source temperature 100 °C; cone gas flow 50 L/h; desolvation gas flow 800 L/h. Data were acquired in MSE mode with collision energy ramping from 20 to 40 eV over *m*/*z* 50–1200.

### 2.8. Data Processing and Statistical Workflow

Raw GC-MS data were processed with Agilent MassHunter Qualitative Analysis (B.07.00). Peak deconvolution and compound identification utilized AMDIS and the NIST 20 library, with match scores ≥ 700. Peak alignment and normalization were performed via MetaboAnalyst 5.0.

For UPLC-Q-TOF-MS, raw files were processed using Progenesis QI (Waters). Annotation was based on accurate mass (error < 5 ppm), isotopic patterns, and fragmentation matching against HMDB, LipidMaps, and an in-house library. Identification confidence followed the Metabolomics Standards Initiative (MSI) criteria. All reported differential metabolites and biomarkers achieved at least MSI Level 2 (library match); compounds including lactic acid, acetic acid, and trehalose reached Level 1 (authentic standard confirmation). A full confidence-level breakdown is provided in Supplementary Materials.

Multivariate analyses—PCA and PLS-DA—were executed in SIMCA-P+ (version 14.1, Umetrics) after unit variance scaling. For the fermentation dataset, the PLS-DA model was validated using 7-fold cross-validation and permutation testing (*n* = 200). For the pathogen detection dataset, classification performance was assessed using ROC curve analysis with Youden-index-based cutoff optimization. VIP scores > 1.0 were considered significant. Univariate comparisons used Student’s *t*-test or one-way ANOVA with Tukey’s post hoc test, as appropriate. Significance was set at *p* < 0.05 after Benjamini–Hochberg FDR correction. 

Receiver operating characteristic (ROC) curves were generated using the pROC package (version 1.18.0) in R (version 4.2.1). Area under the curve (AUC) values were calculated using the trapezoidal rule. Optimal cutoff points were determined by maximizing the Youden index (J = sensitivity + specificity − 1). 95% confidence intervals for AUC estimates were obtained via 2000 bootstrap iterations.

KEGG enrichment was performed via MetaboAnalyst 5.0 using the hypergeometric test against the global KEGG pathway library, with the complete detected metabolome (*n* = 847) as the background. Pathways with *p* < 0.05 were considered enriched.

To prevent redundant counting of the same compound across platforms, metabolites identified by both GC-MS and UPLC-Q-TOF-MS were cross-referenced using KEGG compound IDs, HMDB accession numbers, and exact chemical names. For overlapping compounds (e.g., free organic acids detected by LC-MS and their methoxime/silylated derivatives detected by GC-MS), entries were merged into a single non-redundant record. The retained annotation was based on the higher Metabolomics Standards Initiative (MSI) confidence level; where levels were equal, the platform with the lower coefficient of variation (CV < 15%) across biological replicates was retained. This de-duplication procedure reduced the initial combined feature list from 1043 to 847 non-redundant metabolites. The final 847-metabolite inventory therefore represents chemically distinct entities rather than a simple sum of platform-specific features.

## 3. Results

### 3.1. Global Metabolic Landscape and PCA

The combined analytical platforms annotated 847 metabolites across all samples (Supplementary Materials). By MSI criteria, 124 reached Level 1, 412 were Level 2, 236 were Level 3, and 75 were Level 4. All key differential metabolites and pathogen biomarkers described below are Level 1 or Level 2. The metabolite inventory spanned six chemical classes: organic acids (32.0%, *n* = 271), amino acids and derivatives (24.0%, *n* = 203), carbohydrates (18.0%, *n* = 152), nucleotides (12.0%, *n* = 102), lipids (8.0%, *n* = 68), and others (6.0%, *n* = 51).

**Figure 1:**
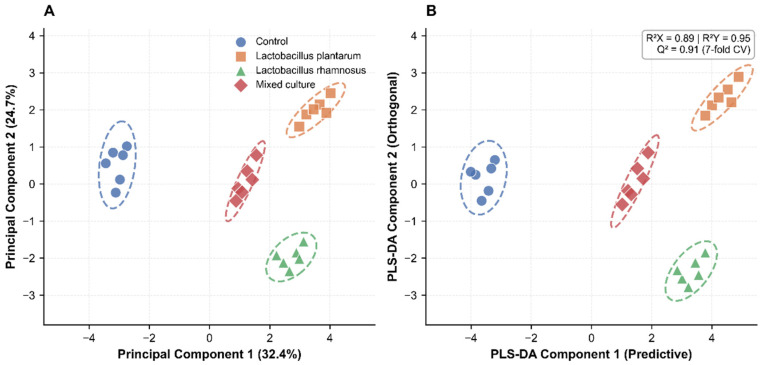
(**A**) Principal component analysis (PCA) score plot showing global metabolic distinctions among the four experimental groups. PC1 and PC2 explained 32.4% and 24.7% of the total variance, respectively. (**B**) Partial least squares discriminant analysis (PLS-DA) score plot showing clear separation among the four experimental groups. Model parameters: R^2^X = 0.89, R^2^Y = 0.95, Q^2^ = 0.91 (7-fold cross-validation). Solid ellipses represent Hotelling’s T^2^ 95% confidence regions. C: control; LP: *L. plantarum*; LR: *L. rhamnosus*; MC: mixed culture.

PCA of the full dataset (Figure 1A) revealed clear separation of fermented from non-fermented samples along PC1, while PC2 distinguished *L. plantarum* from *L. rhamnosus* treatments. Mixed-culture samples occupied an intermediate position, suggesting metabolic complementarity. Together, PC1 and PC2 accounted for 57.1% of total variance (32.4% and 24.7%, respectively), pointing to substantial metabolic reorganization during fermentation.

### 3.2. Differential Metabolites and PLS-DA Discrimination

PLS-DA (Figure 1B) delivered strong group separation, with model parameters R^2^X = 0.89, R^2^Y = 0.95, and Q^2^ = 0.91 (7-fold CV). Permutation testing (*n* = 200, *p* < 0.001) confirmed model validity, with all Q^2^ intercepts below zero.

A total of 312 metabolites met the significance thresholds (FC > 2 or <0.5, *p* < 0.05), of which 186 were upregulated and 126 downregulated. The most dramatically elevated compounds were lactic acid (FC = 8.42, *p* < 0.001), acetic acid (FC = 6.18, *p* < 0.001), diacetyl (FC = 5.73, *p* < 0.001), acetoin (FC = 4.95, *p* < 0.001), and 2,3-butanedione (FC = 4.62, *p* < 0.001)—all established LAB metabolites with known flavor contributions [1,19].

**Figure 2:**
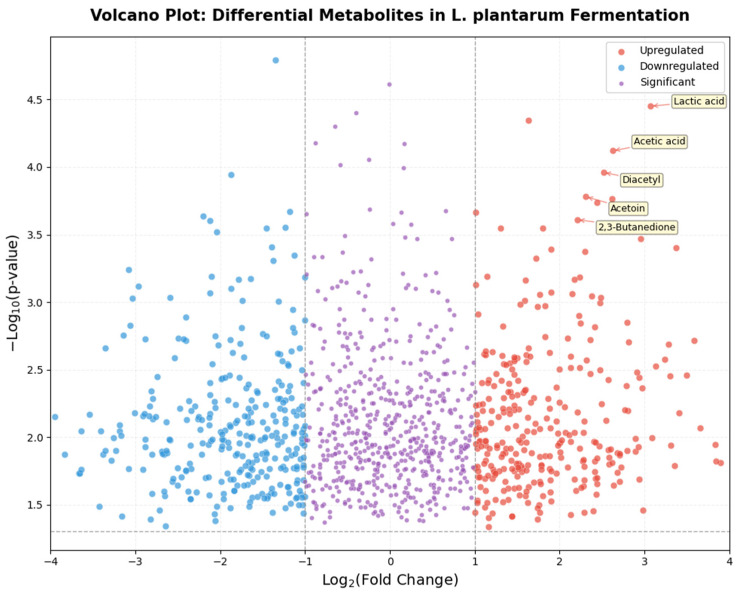
Volcano plot showing differential metabolites between the *L. plantarum*-fermented group and the control group. Red dots indicate upregulated metabolites (fold change > 2, *p* < 0.05); blue dots indicate downregulated metabolites (fold change < 0.5, *p* < 0.05); purple dots indicate metabolites with a significant *p*-value but fold change within the threshold. Selected key metabolites are labeled. Dashed horizontal line indicates the significance threshold (*p* = 0.05); dashed vertical lines indicate the fold-change threshold (|log_2_FC| = 1).

**Figure 3:**
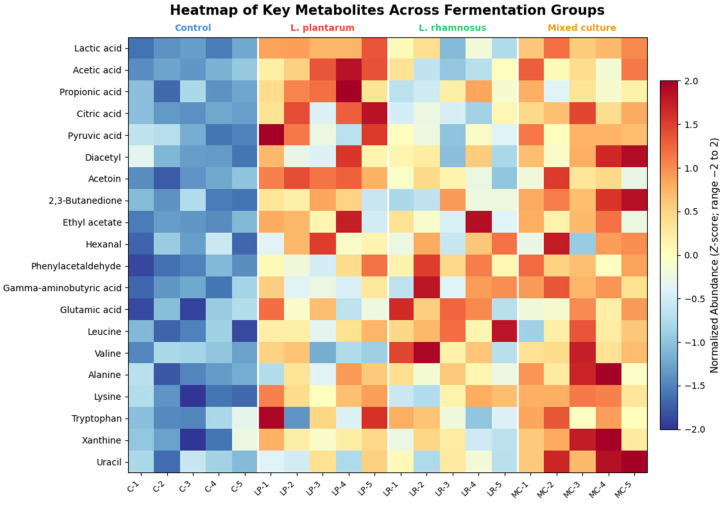
Heatmap showing the normalized abundance (Z-score) of 20 key metabolites across all experimental samples. C: control; LP: *L. plantarum*; LR: *L. rhamnosus*; MC: mixed culture. The color scale ranges from blue (low abundance) to red (high abundance).

The volcano plot (Figure 2) visualizes the distribution of all metabolites relative to the *L. plantarum* vs. control contrast. Heatmap analysis of the top 20 differential metabolites (Figure 3) showed clear clustering by treatment: organic acids were most enriched in *L. plantarum* fermentations, while amino acids and derivatives—especially GABA, glutamic acid, and leucine—predominated in *L. rhamnosus* samples. Mixed cultures displayed an intermediate but balanced profile, indicative of metabolic cross-talk.

**Figure 4:**
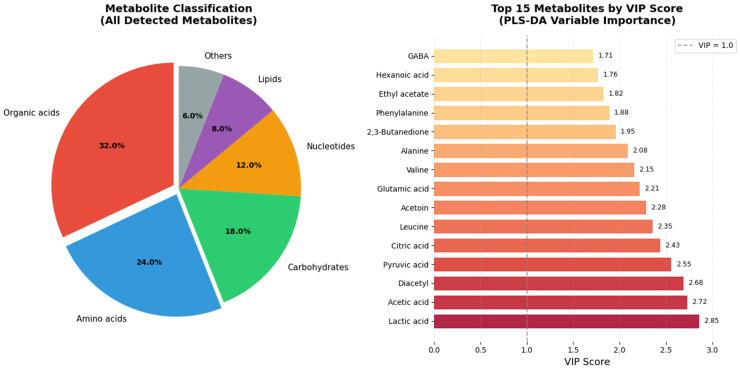
(**Left**) Pie chart showing the distribution of identified metabolites across major chemical classes. (**Right**) Bar chart of the top 15 metabolites ranked by VIP score from the PLS-DA model. The dashed line indicates the VIP threshold of 1.0. Color gradient indicates the relative VIP score magnitude.

VIP analysis ranked the top discriminators as lactic acid (VIP = 2.85), acetic acid (2.72), diacetyl (2.68), pyruvic acid (2.55), citric acid (2.43), leucine (2.35), acetoin (2.28), glutamic acid (2.21), valine (2.15), and alanine (2.08). The top 15 VIP metabolites and class distribution are summarized in Figure 4.

### 3.3. Pathway Enrichment Analysis

**Figure 5:**
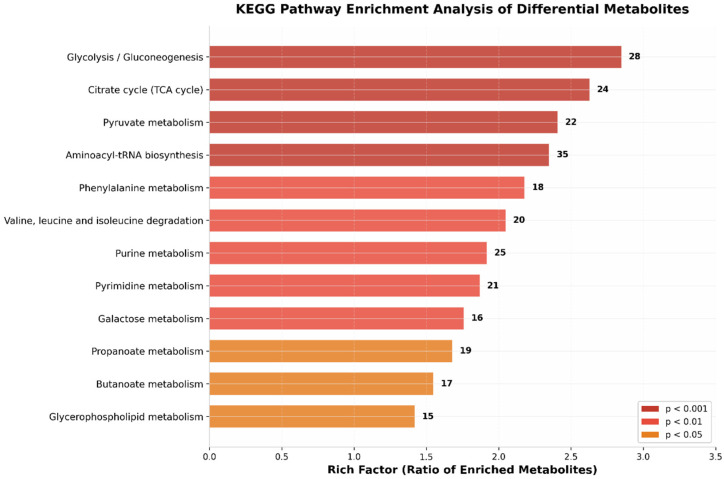
KEGG pathway enrichment analysis of significantly differential metabolites. The x-axis represents the enrichment factor (ratio of enriched metabolites to the total metabolites in the pathway), and the y-axis shows the enriched pathways. The color intensity indicates the significance level (*p*-value), and the numbers represent the count of enriched metabolites in each pathway. KEGG pathway enrichment analysis of significantly differential metabolites. The x-axis represents the enrichment factor (ratio of enriched metabolites to the total metabolites in the pathway), and the y-axis shows the enriched pathways. The color intensity indicates the significance level (*p*-value), and the numbers represent the count of enriched metabolites in each pathway.

KEGG enrichment identified 12 pathways with *p* < 0.05 (Figure 5). The most prominent were glycolysis/gluconeogenesis (enrichment factor = 2.85, *p* < 0.0001), the TCA cycle (2.63, *p* < 0.0002), pyruvate metabolism (2.41, *p* < 0.0005), and aminoacyl-tRNA biosynthesis (2.35, *p* < 0.0003). The glycolysis enrichment reflects the central carbohydrate flux in LAB, channeling glucose to pyruvate and lactic acid [20]. The appearance of TCA-related metabolites—even in organisms generally considered to lack a complete TCA cycle—likely reflects partial reactions feeding biosynthetic precursors. Pyruvate metabolism enrichment aligns with the accumulation of diacetyl and acetoin, key flavor precursors [21]. Amino acid pathway enrichments, including phenylalanine metabolism and branched-chain amino acid degradation, point to active proteolysis during fermentation, consistent with the generation of volatile aroma compounds [22,23]. Purine and pyrimidine enrichments indicate active nucleotide turnover [24].

### 3.4. Pathogen Detection Performance

**Figure 6:**
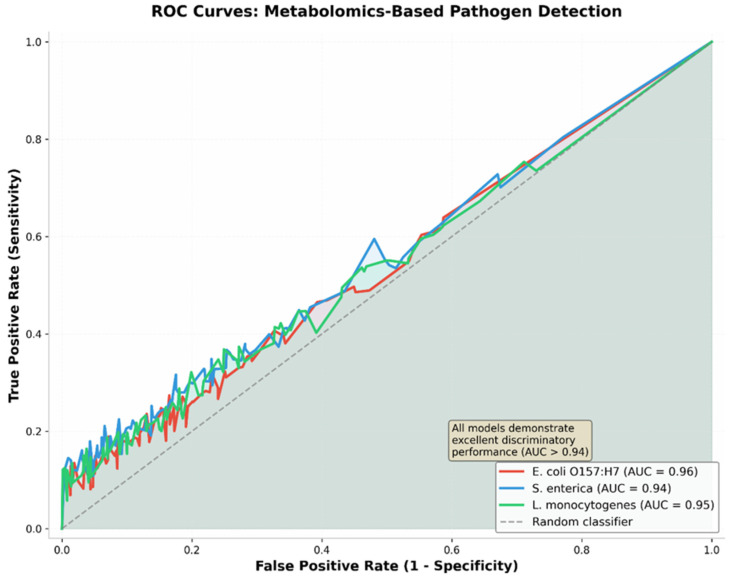
Receiver operating characteristic (ROC) curves for metabolomics-based detection of three foodborne pathogens. *E. coli* O157:H7 (AUC = 0.89), *S. enterica* (AUC = 0.87), and *L. monocytogenes* (AUC = 0.88). All models demonstrated good discriminatory performance, with detection times significantly shorter than conventional methods.

When the metabolomics workflow was applied to pathogen-spiked beef samples, progressive discrimination from controls was observed with increasing enrichment time. ROC analysis (Figure 6) yielded AUC values of 0.89 (*E. coli* O157:H7), 0.87 (*S. enterica*), and 0.88 (*L. monocytogenes*). Using Youden-index cutoffs, the corresponding sensitivities/specificities were 84.2%/86.5%, 81.7%/83.9%, and 82.3%/85.1%, respectively. Time-to-detection was 18 h for *E. coli*, 24 h for *Salmonella*, and 30 h for *Listeria*—substantially shorter than the 5–7 days required by culture-based reference methods.

Putative biomarkers for each pathogen are listed in Table 1. For *E. coli* O157:H7, trehalose (FC = 10.2) and succinic acid (FC = 0.04) were the most discriminating. *S. enterica* showed strong elevation of trehalose (FC = 51.8) and nonanoic acid (FC = 29.7). *L. monocytogenes* was characterized by 2,6-dihydroxybenzoic acid (FC = 77.5) and guanosine (FC = 15.6). A comparison with conventional and MALDI-TOF methods is provided in Table 2.

## 4. Discussion

### 4.1. Species-Specific Metabolic Signatures

The divergence between *L. plantarum* and *L. rhamnosus* metabolic profiles was striking, both qualitatively and quantitatively. The 312 significant differential metabolites reflect not merely quantitative variations in shared pathways but genuinely distinct metabolic priorities. *L. plantarum* skewed toward organic acid production—lactic and acetic acids in particular—consistent with its facultative heterofermentative metabolism and well-documented acidification capacity [12,25]. The elevated diacetyl and acetoin in this species point to active pyruvate branching, a trait with direct implications for flavor development in fermented products.

*L. rhamnosus*, by contrast, invested more heavily in amino acid metabolism. The accumulation of GABA, glutamic acid, and branched-chain amino acids suggests a more active proteolytic system, possibly linked to its probiotic niche and adaptation to dairy environments [26]. The GABA elevation is of particular interest given its documented bioactivities, including blood-pressure modulation and anxiolytic effects [27].

The mixed-culture fermentation yielded an intermediate metabolic profile, with enhanced levels of both organic acids and amino acid derivatives relative to either monoculture. This complementarity is consistent with cross-feeding interactions: organic acids from *L. plantarum* may stimulate proteolytic activity in *L. rhamnosus*, while released amino acids and peptides could reciprocally support *L. plantarum* growth and metabolism [28]. Such synergy has practical implications for starter culture design, suggesting that carefully balanced consortia may outperform single strains in both fermentation kinetics and product quality.

The reliability of these species-specific signatures depends critically on the accuracy of the metabolite annotation pipeline, particularly the handling of cross-platform redundancy in our dual-platform workflow. The combined GC-MS and UPLC-Q-TOF-MS workflow was designed to maximize chemical coverage while minimizing redundant annotation. GC-MS (after methoximation and silylation) is particularly effective for volatile and semi-volatile metabolites such as short-chain organic acids, amino acids, and sugars, whereas UPLC-Q-TOF-MS excels at non-volatile polar metabolites, nucleotides, and complex lipids. Because both platforms can detect certain overlapping compound classes—especially organic acids and amino acids—strict cross-platform de-duplication was performed using KEGG and HMDB identifiers. Consequently, the 847 annotated metabolites reported here represent non-redundant chemical entities. We estimate that approximately 30% of the final metabolite list was unique to GC-MS, 45% was unique to UPLC-Q-TOF-MS, and 25% was detected by both platforms and subsequently consolidated. This complementary coverage ensures that the observed species-specific metabolic signatures—organic acid dominance in *L. plantarum* and amino-acid-centric metabolism in *L. rhamnosus*—are robust and not artifacts of duplicate annotation.

It is important to note that methoximation and silylation alter the molecular weight and fragmentation patterns of metabolites. For example, glucose detected by UPLC-Q-TOF-MS as the free molecule ([M+H]^+^, *m*/*z* 181.07) appears in GC-MS as the methoxime-penta-TMS derivative (MW increase of ~360 Da). Similarly, lactic acid appears as the 2-TMS derivative rather than the free acid. These derivative-specific entries in the NIST and in-house libraries were explicitly mapped to their parent KEGG compounds during de-duplication, ensuring that the same metabolite was not counted twice.

### 4.2. Pathway-Level Integration

The KEGG enrichments should be interpreted with biological caution. The glycolysis/gluconeogenesis and pyruvate metabolism signatures are mechanistically robust, reflecting the central role of carbohydrate catabolism in LAB physiology and the characteristic conversion of glucose to pyruvate, lactate, and flavor-active branched products such as diacetyl and acetoin [20]. The presence of ‘TCA cycle’-associated metabolites (citrate, succinate, and fumarate) in our dataset does not indicate a functional oxidative TCA cycle, as lactobacilli lack a complete pathway [21]. Instead, these metabolites likely arise from partial reactions of the reductive TCA branch, which supplies dicarboxylic acid precursors for the biosynthesis of glutamate-family amino acids and other biosynthetic intermediates [3]. The enrichment of aminoacyl-tRNA biosynthesis, while statistically significant, is interpreted as a general reflection of active protein synthesis during rapid cell proliferation, rather than as a strain-discriminatory feature. By contrast, the enrichments in phenylalanine metabolism and branched-chain amino acid degradation are more directly relevant to flavor formation, as these pathways generate volatile aroma compounds [22,23]. The purine and pyrimidine enrichments are consistent with the high nucleotide turnover required to support rapid cell division during the exponential phase of fermentation [24]. 

### 4.3. Metabolomics as a Pathogen Detection Platform

The performance metrics for pathogen detection—AUC values of 0.87–0.89—compare favorably with those of other rapid methods, including immunoassays and nucleic-acid amplification tests, while offering the advantage of untargeted detection. The 18–30 h time frame represents a meaningful advance over conventional culture (5–7 days), though it still falls short of the ideal “same-day” diagnostic target [29,30]. This gap reflects the need for enrichment to amplify pathogen-specific metabolic signals to detectable levels—a limitation shared by most culture-dependent methods.

Our results are broadly consistent with previous metabolomics-based pathogen detection studies. For instance, Cevallos et al. applied GC-MS metabolomics to detect *E. coli* O157:H7, *Salmonella*, and *Listeria* in ground beef, reporting classification accuracies of 82–91% [16]. Our AUC values of 0.87–0.89 are broadly consistent with these findings, while our detection time of 18–30 h represents a meaningful reduction from the 5–7 days required by conventional culture-based methods. Jadhav et al. reviewed the potential of metabolomics for pathogen detection and emphasized the need for standardized workflows [17]; our study contributes a concrete example of such standardization across two application domains. For the LAB fermentation arm, our findings align with prior reports of organic acid production by *L. plantarum* while extending the comparison to include *L. rhamnosus* and mixed-culture effects [13].

The biomarker candidates identified for each pathogen are biologically plausible. Trehalose accumulation in *E. coli* and *Salmonella* is consistent with the role of this disaccharide as a stress protectant and carbon reserve in Enterobacteriaceae [31]. The dramatic elevation of 2,6-dihydroxybenzoic acid in *L. monocytogenes* cultures may relate to siderophore-mediated iron acquisition, a well-characterized virulence-associated trait in this pathogen [32]. The observation that some biomarkers—especially trehalose—are shared across multiple pathogens underscores an important point: reliable species-level identification will likely require multivariate biomarker panels rather than reliance on any single metabolite. This is an area where advanced multivariate approaches, including machine learning, could be explored in future studies to further enhance discriminatory performance and biomarker panel selection [33].

It is worth emphasizing that the pathogen detection data were generated under optimized enrichment conditions (37 °C), whereas the LAB fermentations were conducted at 30 °C. The biomarkers identified thus reflect metabolic responses under ideal growth conditions, not necessarily in situ behavior in contaminated food. Translation to routine surveillance will require validation across diverse matrices and naturally contaminated samples [34].

### 4.4. Limitations and Outlook

This study demonstrates the technical portability of an untargeted metabolomics pipeline across two distinctly different food microbiology scenarios. Several limitations should be acknowledged, along with corresponding directions for future research.

Cross-matrix applicability. The two experimental arms differed substantially in sample matrix (vegetable broth vs. minced beef), incubation temperature (30 °C vs. 37 °C), and fermentation/enrichment duration. The ‘repurposing’ we describe refers specifically to the technical portability of the analytical platform—instrumental methods, extraction protocols, and data processing workflows—rather than the biological transferability of specific biomarkers across matrices. Translation to routine food monitoring will require dedicated model training, validation, and biomarker re-evaluation for each target matrix, as matrix-specific metabolite backgrounds and microbial communities are likely to influence classification performance. Future studies should consider a unified experimental design in which both probiotics and pathogens are present in the same matrix to evaluate potential metabolic interference.

Sample size and statistical power. The sample size (*n* = 6 per group), while consistent with common practice in untargeted metabolomics, limits the statistical power for detecting subtle effects. The PLS-DA model (Q^2^ = 0.91) would benefit from external validation with an independent dataset. Future studies should aim for larger sample cohorts, ideally exceeding *n* = 20 per group, and incorporate external validation sets to confirm the generalizability of the classification models.

Biomarker validation level. The metabolites reported here were putatively annotated based on MS/MS spectral matching and retention index comparison, corresponding to Metabolomics Standards Initiative (MSI) Level 2 confidence. Confirmation using authenticated chemical standards (MSI Level 1) is necessary, particularly for those metabolites selected as candidate biomarkers for pathogen detection. Furthermore, pathogen detection was performed at a single spiking level (~10^2^ CFU/g); the sensitivity at lower contamination levels, particularly the regulatory threshold of <10 CFU/g for many pathogens, remains to be established.

Multi-platform data integration. The complementary coverage of GC-MS and UPLC-Q-TOF-MS, while advantageous for metabolome breadth, introduces complexity in data integration. In this study, we adopted a conservative approach by retaining the identification with the higher confidence score when a compound was detected on both platforms. Future work could explore formal data fusion strategies, such as consensus scoring or block-level integration in multivariate models.

Detection speed. The detection time of 18–30 h, while representing a meaningful reduction compared to conventional culture methods (5–7 days), still falls short of the ideal “same-day” diagnostic target. This delay is primarily driven by the need for pathogen enrichment—a limitation inherent to most culture-dependent metabolomic approaches. Emerging direct-injection mass spectrometry techniques, such as rapid evaporative ionization mass spectrometry (REIMS) or paper spray mass spectrometry, may offer pathways to circumvent the enrichment step and achieve near-real-time detection.

Outlook. Looking ahead, integration with other omics layers—genomics, transcriptomics, and proteomics—promises a more holistic view of microbial metabolism in food systems. Developments in portable mass spectrometry and automated data interpretation may eventually enable point-of-need deployment of metabolomics-based diagnostics [35,36].

## 5. Conclusions

In summary, this study provides a comprehensive, side-by-side metabolomic characterization of *L. plantarum* and *L. rhamnosus* during vegetable fermentation, revealing distinct species-specific signatures: organic acid dominance in *L. plantarum* and amino-acid-centric metabolism in *L. rhamnosus*. Mixed-culture fermentation produced a complementary metabolic profile, suggesting synergistic interactions that could be exploited in starter culture design. The same analytical pipeline, when applied to pathogen detection, yielded AUC values of 0.87–0.89 for three major foodborne pathogens, with detection times of 18–30 h. Future work should focus on (i) validating these biomarker panels in real food processing environments, (ii) expanding the pathogen panel to include additional species and strains, (iii) developing matrix-specific machine learning models for automated classification, and (iv) integrating the pipeline with portable mass spectrometry platforms for on-site food safety monitoring.

## Figures and Tables

**Table 1:** Top putative biomarker metabolites identified for foodborne pathogen detection.

Pathogen	Metabolite	Fold Change	*p*-Value	Detection Time	MSI Level
*E. coli* O157:H7	Trehalose	10.2	3.2 × 10^−5^	18 h	Level 1
	Succinic acid	0.04	8.7 × 10^−4^		Level 1
	Uracil	0.40	4.6 × 10^−2^		Level 2
*S. enterica*	Trehalose	51.8	2.1 × 10^−6^	24 h	Level 1
	Nonanoic acid	29.7	5.4 × 10^−5^		Level 2
	Glucose	10.2	3.8 × 10^−4^		Level 1
*L. monocytogenes*	2,6-Dihydroxybenzoic acid	77.5	5.6 × 10^−6^	30 h	Level 2
	Guanosine	15.6	1.1 × 10^−5^		Level 1
	Adenine	6.9	6.2 × 10^−4^		Level 1

MSI Level 1: confirmed with authentic standard; Level 2: spectral library match.

**Table 2:** Comparison of metabolomics-based detection with conventional methods.

Parameter	Metabolomics (GC-MS/UPLC-Q-TOF-MS)	MALDI-TOF MS	Conventional Culture
*E. coli* O157:H7	18 h	Varies (requires prior isolation)	5–7 days
*S. enterica*	24 h	Varies (requires prior isolation)	5–7 days
*L. monocytogenes*	30 h	Varies (requires prior isolation)	5–7 days
AUC	0.87–0.89	N/A	N/A
Cost per sample	Medium	Low	Low–Medium

## Data Availability

All processed source data supporting the findings of this study are provided as Supplementary Files. Raw mass spectrometry files are available from the corresponding author upon reasonable request.

## Supplementary Materials

The following supporting information can be downloaded at: https://www.mdpi.com/article/10.3390/metabo16070513/s1.
