# Supplementary material for: Untargeted Metabolomics Reveals Distinct Metabolic Signatures of Lactic Acid Bacteria in Food Fermentation and the Same Pipeline Applied to Foodborne Pathogen Detection
Source: Metabolites. 2026 Jul 22;16(7):513. doi: 10.3390/metabo16070513 (PMC13413759; doi:10.3390/metabo16070513)
Supplement: Supplementary file 1 [file metabolites-16-00513-s001.zip › Supplementary Figure S1.pdf]

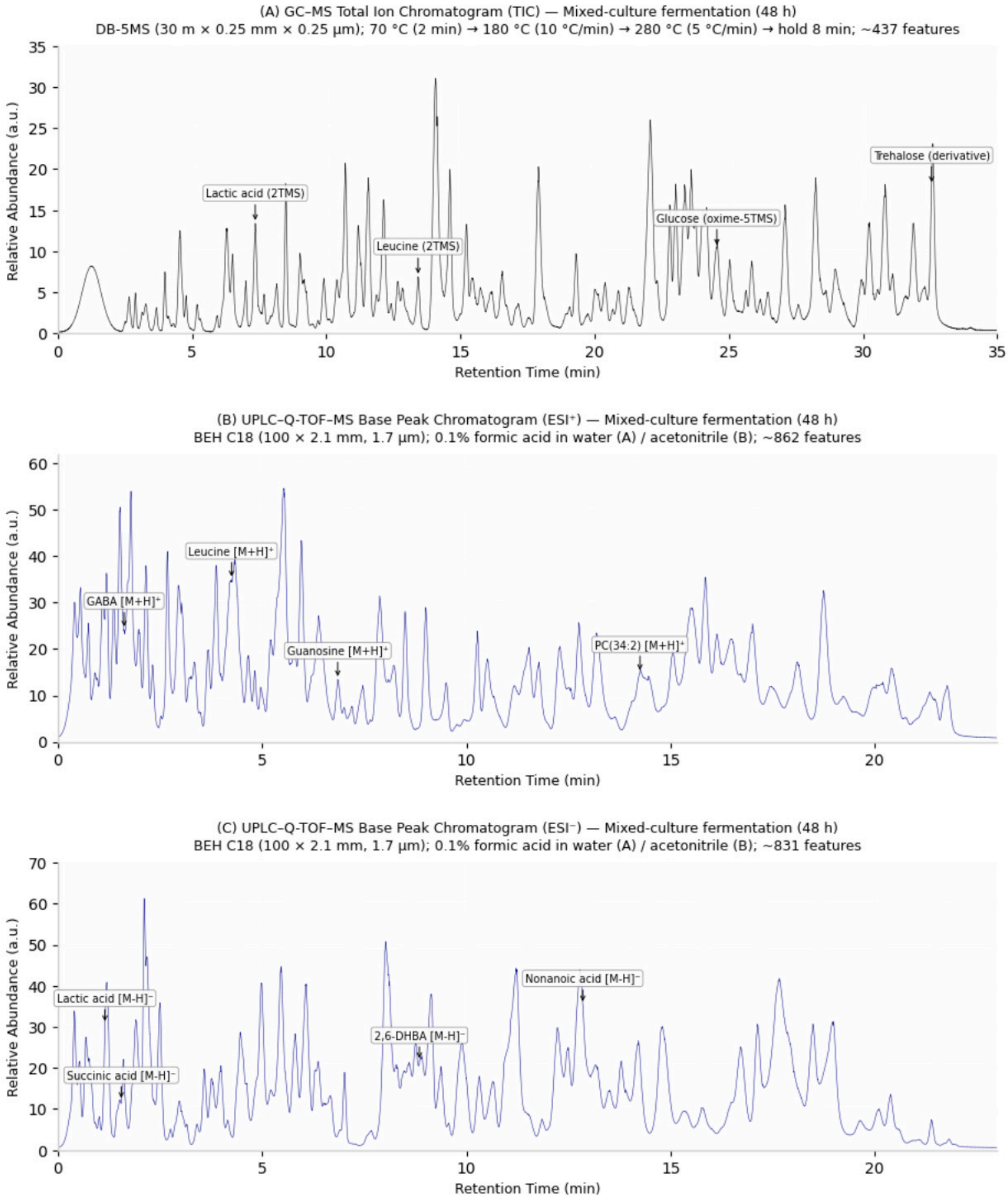

Supplementary Figure S1. Representative chromatograms from the multi-platform untargeted metabolomics workflow. (A) GC–MS total ion chromatogram (TIC) of a mixed-culture fermentation sample (48 h, *L. plantarum* + *L. rhamnosus*). The sample was extracted with methanol:chloroform:water (2.5:1:1, v/v/v), derivatized by methoximation (20 mg/mL methoxyamine hydrochloride in pyridine, 37 °C, 90 min) followed by silylation with MSTFA (37 °C, 30 min), and analyzed on an Agilent 7890B/5977B system equipped with a DB-5MS capillary column (30 m × 0.25 mm ×

0.25 μm). The temperature program was: 70 °C (2 min hold) → 180 °C at 10 °C/min → 280 °C at 5 °C/min → hold 8 min. Approximately **437 features** were deconvoluted by AMDIS and annotated against the NIST 20 library (match score ≥ 700). Representative annotated peaks include lactic acid (2-TMS derivative, ~7.4 min), leucine (2-TMS derivative, ~13.4 min), glucose (methoxime + 5-TMS derivative, ~24.6 min), and trehalose (derivative, ~32.6 min). **(B)** UPLC–Q-TOF–MS base peak chromatogram (BPC) in positive electrospray ionization (ESI^+) mode of the same mixed-culture sample. Chromatographic separation was achieved on an ACQUITY UPLC BEH C18 column (100 mm × 2.1 mm, 1.7 μm) at 40 °C with a mobile phase consisting of 0.1% formic acid in water (A) and acetonitrile (B) at 0.4 mL/min. The gradient was: 0–2 min, 5% B; 2–10 min, 5–40% B; 10–15 min, 40–70% B; 15–18 min, 70–95% B; 18–20 min, 95% B; 20–23 min, 5% B. Approximately **862 features** were detected after peak picking and alignment in Progenesis QI. Representative annotated peaks include GABA ($[M+H]^+$, ~1.6 min), leucine ($[M+H]^+$, ~4.3 min), guanosine ($[M+H]^+$, ~6.9 min), and phosphatidylcholine PC(34:2) ($[M+H]^+$, ~14.3 min). **(C)** UPLC–Q-TOF–MS BPC in negative electrospray ionization (ESI^-) mode, showing complementary detection of organic acids and fatty acids. Approximately **831 features** were detected in negative mode. Representative annotated peaks include lactic acid ($[M-H]^-$, ~1.2 min), succinic acid ($[M-H]^-$, ~1.6 min), 2,6-dihydroxybenzoic acid ($[M-H]^-$, ~8.9 min), and nonanoic acid ($[M-H]^-$, ~12.9 min).
